# Inequity of antenatal influenza and pertussis vaccine coverage in Australia: the Links2HealthierBubs record linkage cohort study, 2012–2017

**DOI:** 10.1186/s12884-023-05574-w

**Published:** 2023-05-08

**Authors:** Lisa McHugh, Annette K Regan, Mohinder Sarna, Hannah C Moore, Paul Van Buynder, Gavin Pereira, Christopher C Blyth, Karin Lust, Ross M Andrews, Kristy Crooks, Peter Massey, Michael J Binks

**Affiliations:** 1 School of Public Health, Division of Medicine, University of Queensland, Brisbane, Qld 4001 Australia; 2 Curtin School of Population Health, Curtin University, Perth, WA Australia; 3 School of Nursing and Health Professions, University of San Francisco, San Francisco, CA USA; 4 Wesfarmers Centre for Vaccines & Infectious Diseases, Telethon Kids Institute, Perth, WA Australia; 5 School of Medicine and Dentistry, Griffith University, Southport, QLD Australia; 6 enAble Institute, Curtin University, Perth, WA Australia; 7 School of Medicine, The University of Western Australia, Perth, WA Australia; 8 Department of Paediatric Infectious Diseases, Perth Children’s Hospital, Perth, WA Australia; 9 Department of Microbiology, PathWest Laboratory Medicine, Perth, WA Australia; 10 Women’s and Newborn Service, Royal Brisbane and Women’s Hospital, Brisbane, QLD Australia; 11 Department of Medicine, The University of Queensland, Brisbane Queensland, Australia; 12 Australian National University Canberra, Canberra, Australia; 13 Menzies School of Health Research, Charles Darwin University, Darwin, NT Australia; 14 College of Medicine and Dentistry, James Cook University, Queensland, Australia; 15 Centre for Fertility and Health, Norwegian Institute of Public Health, Oslo, Norway

**Keywords:** Inequity, Antenatal, Influenza, Pertussis, Vaccination, Pregnancy, Australia, First Nations

## Abstract

**Background:**

Pregnancy and early infancy are increased risk periods for severe adverse effects of respiratory infections. Aboriginal and/or Torres Strait Islander (respectfully referred to as First Nations) women and children in Australia bear a disproportionately higher burden of respiratory diseases compared to non-Indigenous women and infants. Influenza vaccines and whooping cough (pertussis) vaccines are recommended and free in every Australian pregnancy to combat these infections. We aimed to assess the equity of influenza and/or pertussis vaccination in pregnancy for three priority groups in Australia: First Nations women; women from culturally and linguistically diverse (CALD) backgrounds; and women living in remote areas or socio-economic disadvantage.

**Methods:**

We conducted individual record linkage of Perinatal Data Collections with immunisation registers/databases between 2012 and 2017. Analysis included generalised linear mixed model, log-binomial regression with a random intercept for the unique maternal identifier to account for clustering, presented as prevalence ratios (PR) and 95% compatibility intervals (95%CI).

**Results:**

There were 445,590 individual women in the final cohort. Compared with other Australian women (n = 322,848), First Nations women (n = 29,181) were less likely to have received both recommended antenatal vaccines (PR 0.69, 95% CI 0.67–0.71) whereas women from CALD backgrounds (n = 93,561) were more likely to have (PR 1.16, 95% CI 1.10–1.13). Women living in remote areas were less likely to have received both vaccines (PR 0.75, 95% CI 0.72–0.78), and women living in the highest areas of advantage were more likely to have received both vaccines (PR 1.44, 95% CI 1.40–1.48).

**Conclusions:**

Compared to other groups, First Nations Australian families, those living in remote areas and/or families from lower socio-economic backgrounds did not receive recommended vaccinations during pregnancy that are the benchmark of equitable healthcare. Addressing these barriers must remain a core priority for Australian health care systems and vaccine providers. An extension of this cohort is necessary to reassess these study findings.

**Supplementary Information:**

The online version contains supplementary material available at 10.1186/s12884-023-05574-w.

## Introduction

Pregnancy and early infancy are increased risk periods for severe adverse effects of respiratory infections [1, 2]. A decrease in immunity from physiological effects of pregnancy, [3] and age-associated immune immaturity in infants, exacerbated in infants born preterm, are among the reasons for these heightened infection risks [2]. Other risk factors include co-morbidities, living in remote regions, lower socio-economic status, inadequate housing, decreasing rates of exclusive breastfeeding, and limited access to culturally safe and appropriate, affordable health care [4]. To combat respiratory infections among these groups, inactivated influenza vaccines (IIV) and pertussis-antigen containing vaccines (dTpa) are recommended during pregnancy and provided free to all Australian women, [5] however data to inform the equity of antenatal vaccination coverage are scarce.

Internationally, antenatal IIV and dTpa vaccination studies have reported a lower uptake of both vaccines among their Indigenous populations compared to uptake in Asian/European ethnicities [6–8]. Aboriginal and/or Torres Strait Islander (herein respectfully referred to as “First Nations”) women and children in Australia bear a disproportionately high burden of respiratory diseases compared to non-Indigenous women and infants [9]. As such, First Nations women are a priority for antenatal vaccination. Several Australian studies incorporating First Nations women have suggested the coverage of IIV in pregnancy is low (range < 3–49%), however small samples sizes and biases [10–13] precluded reliable estimates of coverage. We previously reported lower and declining proportions of IIV and dTpa vaccination coverage among First Nations pregnant women compared to non-Indigenous pregnant women [14]. Also, although over 30% of Australian women are born overseas, [15] data describing antenatal IIV and dTpa vaccination coverage in women from culturally and linguistically diverse populations (CALD) are scarce. One Australian survey conducted over a three-month period examined IIV and dTpa vaccination coverage in pregnant women from CALD backgrounds in Victoria [16]. Although these results are important, the small sample size (n = 370) and restricted location preclude generalisable estimates of vaccine coverage in CALD women from other Australian jurisdictions and do not allow evaluation of temporal trends. A population-based study using data from Western Australia (WA) and New South Wales examined childhood vaccination coverage and showed pockets of lower vaccine coverage in children born from CALD mothers [17]. It is not known whether these pockets of low vaccine coverage extends to antenatal vaccination.

To our knowledge, no other Australian study has examined the influence of living in a remote area, or the level of socio-economic variation upon IIV and dTpa vaccine coverage in First Nations, non-Indigenous and non-CALD (hereafter referred to as ‘other Australian’), and CALD pregnant women.

The primary aim of this study was to provide robust estimates of antenatal IIV and/or dTpa vaccine coverage across three Australian jurisdictions over six years, among (1) women who identified as First Nations, who were from CALD backgrounds, or other Australians, and (2) among women from different levels of socio-economic advantage/disadvantage and remoteness of living. These data together are essential to indicate the equity of the Australian National Immunisation Program (NIP) and guide future antenatal vaccination policy.

## Methods

### Study design and population

Links2HealthierBubs (Links2HB) is a retrospective observational cohort study of mother-infants pairs [18]. The eligible cohort comprised mothers with a pregnancy ≥ 20 weeks gestation, between 01 Jan 2012-31 and Dec 2017 inclusive, in the Northern Territory (NT), Queensland (Qld), and WA. Collectively these jurisdictions encompass 5.92 million km^2^ of land, incorporating the largest geographic land mass and diverse climatic variations in Australia, [19] with ~ 95,000 births annually, (~ 32% of Australia’s total number of births) [20].

### Inclusions/exclusions

Women were excluded from the study if: they recorded a birth before 20 weeks gestation; had missing data related to gestation at birth; or birthed outside the study period.

### Data collection methods and sources

The cohort was created through the linkage of Perinatal Data Collections (PDCs) and immunisation registers and databases. These data sources are described in Supplementary box 1. A combination of deterministic and probabilistic record linkage was used to identify individual women and their pregnancies. Analysis of antenatal IIV coverage involved the whole study period (2012–2017) whereas dTpa was analysed from 2015 onwards following implementation of funded vaccination programs [21].

The key variables used to estimate vaccination coverage were; infant date of birth, gestation in weeks at the time of infant birth, gestation in weeks at the time of vaccination, and trimester of pregnancy in which maternal vaccination occurred. Vaccination in pregnancy (antenatal vaccination) was defined by documented receipt of an IIV and/or dTpa vaccine between the date of conception and the date of infant birth. Participants with no IIV and/or dTpa vaccination data during a pregnancy were considered ‘unvaccinated in pregnancy’.

#### First Nations status, ethnicity, socio-economic advantage and geographic variation

First Nations status was determined from identifiers located in PDCs and hospital admissions data. In WA, the data linkage branch also identifies Indigeneity through a validated algorithm implemented by the WA Department of Health, for any individual with at least one record in a government administrative dataset [22].

Ethnicity and mother’s country of birth variables recorded in the PDCs were used to categorise women from CALD backgrounds. Country of birth classifications were from Standard Australian Classification of Countries (SACC), 2016 Australian Bureau of Statistics [23]. A composite variable was created to classify women as (a) ‘First Nations’ if they identified as ‘Indigenous’ AND were classified as ‘Aboriginal and/or Torres Strait Islander’ in the ethnicity variable; (b) CALD if they did not identify as First Nations AND were not born in Australia AND were not classified as Caucasian in the ethnicity variable; and (c) ‘Other Australian’ if they were Australian born and did not identify as First Nations AND were classified as ‘Caucasian’ in the ethnicity variable (Supplementary box 2).

The Index of Relative Socio-economic Advantage and Disadvantage (IRSAD); derived from the Socio-Economic Indexes for Australia (SEIFA; Australian Bureau of Statistics 2016) index, [24] was used to define socio-economic advantage and disadvantage. The score includes measures of economic, educational and social domains and is ranked on a scale from 1 to 10, with one being the most disadvantaged and 9 and 10 being the most advantaged. The Accessibility Remoteness Index of Australia [25, 26] was used to identify remote and non-remote living participants, and hence their accessibility to health care services. Remote and very remote categories were combined to enhance statistical power. The mother’s recorded postcode of residence at the time of infant birth was used to establish their SEIFA and ARIA indexes.

### Data analysis

The number of pregnancies, proportion of women who were administered antenatal vaccinations, and socio-economic and geographic characteristics were calculated for First Nations, Other Australian and CALD participants. A binomial generalized linear mixed model (GLMM) with a random intercept for the mother’s ID was used to account for clustering of participants who were in the cohort more than once over the study period. To determine the relative influence of (a) living remotely [25] and (b) socio-economic disadvantage (IRSAD/SEIFA) on antenatal IIV and dTpa vaccine coverage, we then calculated unadjusted prevalence risks (PR) and 95% compatibility intervals (95% CIs) [27]. All potential confounding variables statistically (5% significance level in univariable analysis) or speculatively associated with maternal vaccination coverage were then included in a multivariable GLMM and reported as adjusted prevalence ratios (aPR) with 95% CIs. Not living remotely and SEIFA 1 were used as the reference groups. All data analyses were conducted using Stata statistical software v.17.1 (StataCorp, College Station, Texas).

#### Missing data

Data for the main variables of interest ‘First Nations’ and ‘Ethnicity’ were > 99% complete. Denominators and accompanying proportions for other variables with missing data are presented in tables.

### Ethics and governance

Multi-jurisdictional ethics committee approvals were gained from WA Department of Health (HREC 2016/56), Curtin University (HRE2017-0808), Menzies School of Health Research (HREC 2018–3199), Queensland Health and Royal Brisbane and Women’s Hospital (HREC/2018/QRBW/47,660), and WA Aboriginal Health Ethics Committees (HREC 889) [18].

## Results

**Fig. 1:**
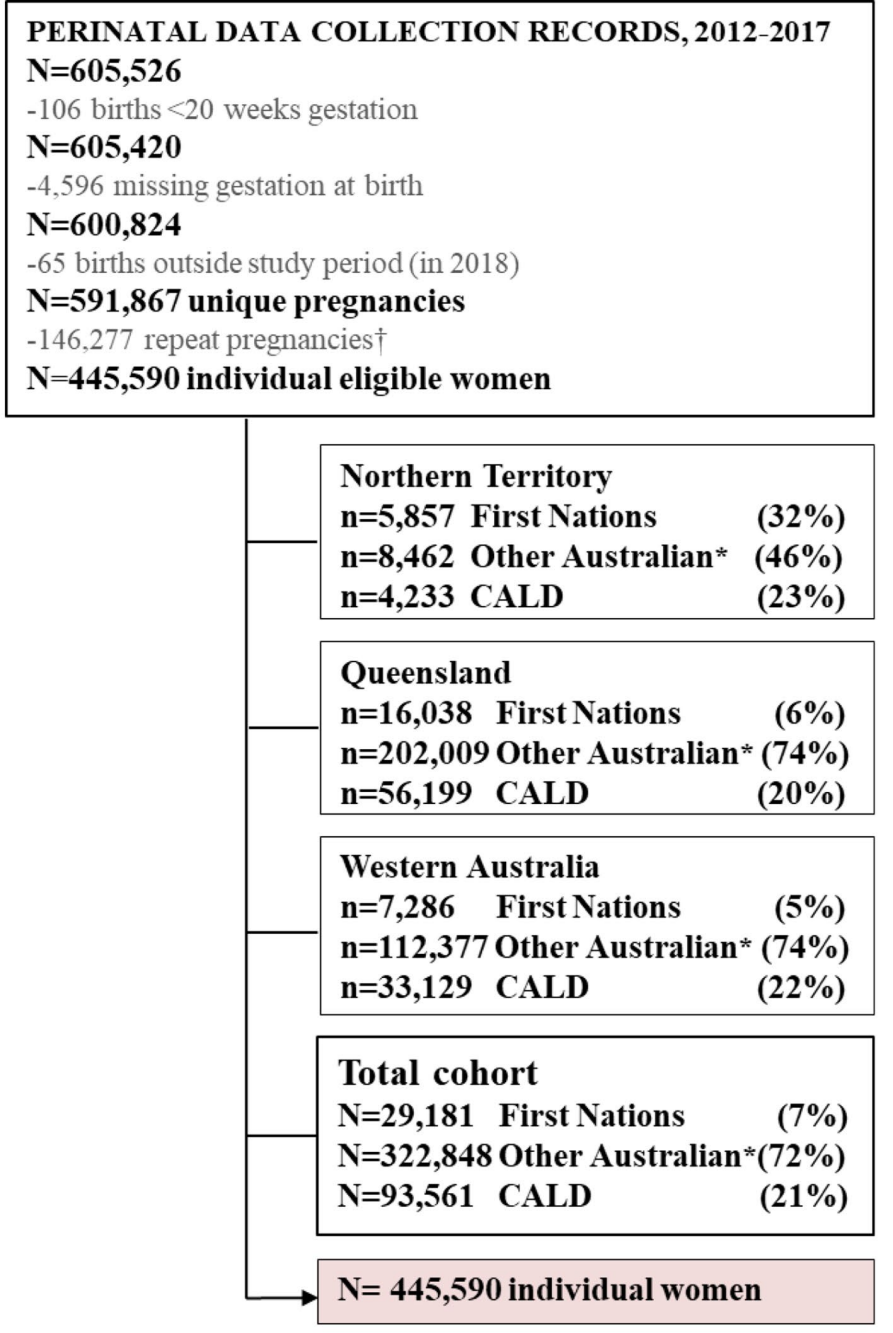
Participant flow diagram of unique Links2HealthierBubs study participants by ethnicity and jurisdiction, Australia, 2012–2017 † Participants within the cohort more than once were identified * Australian born women, who did not identify as First Nations and were classified as ‘Caucasian’ in the variable ‘Ethnicity’

In the final Links2HB cohort (Fig. 1), there were 445,590 individual women out of 591,867 unique pregnancies; comprising 29,181 First Nations, 322,848 Other Australian and 93,561 women from CALD backgrounds.

**Table 1:** Demographic characteristics of LinksHealthierBubs participants by Indigeneity and ethnicity, Australia 2012–2017

Characteristics n (%)	Total N = 445 590	First Nations N = 29 181	Other Australian* N = 322 848	CALD N = 93 561
Maternal age at infant birth < 20 years	17 145 (38)	5 138 (18)	11 239 (38)	768 (1)
20–34 years	330 003 (75)	20 866 (72)	241 167 (75)	67 970 (38)
≥ 35 years	94 595 (21)	2 965 (39)	67 497 (21)	24 133 (26)
Antenatal care in 1st trimester	283 659 (66)	15 191 (54)	207 614 (67)	60 854 (67)
Primiparous	198 348 (45)	10 568 (36)	142 362 (45)	45 418 (49)
Public hospital birth†	174 036/271 737 (64)	14 811/15 912 (93)	129 075/200 084 (65)	30 150/55 741 (54)
Major cities	293 285 (66)	7 467 (26)	210 865 (66)	74 953 (81)
Inner regional	61 453 (14)	3 606 (12)	51 848 (38)	5 999 (38)
Outer regional	64 106 (15)	7 963 (28)	45 890 (14)	10 253 (11)
Remote/Very remote living§	22 912 (5)	9 916 (34)	11 398 (38)	1 598 (2)
***SEIFA scores*** *¶*				
SEIFA score = 1	49 302 (11)	11 866 (41)	28 654 (9)	8 782 (9)
SEIFA score = 2	44 172 (39)	4 309 (15)	32 300 (39)	7 563 (38)
SEIFA score = 3	41 667 (9)	2 962 (39)	30 318 (39)	8 387 (9)
SEIFA score = 4	43 052 (39)	2 492 (9)	32 066 (39)	8 494 (9)
SEIFA score = 5	40 937 (9)	2 101 (38)	31 942 (39)	6 894 (38)
SEIFA score = 6	45 417 (39)	1 737 (38)	33 298 (39)	10 382 (11)
SEIFA score = 7	47 702 (11)	1 191 (38)	36 093 (11)	10 418 (11)
SEIFA score = 8	44 802 (39)	1 099 (38)	34 106 (11)	9 597 (39)
SEIFA score = 9	42 394 (39)	715 (2)	30 216 (9)	11 463 (12)
SEIFA score = 10	40 860 (9)	441 (2)	29 849 (9)	10 570 (11)

**Abbreviations:** CALD, Culturally and linguistically diverse; SEIFA, Socio-Economic Indexes for Australia

* Women who were Australian born, who did not identify as First Nations and were classified as ‘Caucasian’ in the variable ‘Ethnicity’

† Qld data only

§ Based on Accessibility Remoteness Index of Australia

¶ Ranking out of 10, where 1 = highest level of disadvantage, and 10 = most advantaged

Overall, 69,670 (16%) received an IIV during pregnancy, 125,023 (43%) received dTpa and 53,861 (18%) received both vaccines during pregnancy. Respective coverage of IIV among First Nations, Other Australian and CALD women was 14%,15% and 19%; for dTpa this was 31%, 44% and 43%; and for both vaccines, 13%, 18% and 21% (Supplementary Table 1). Compared to other Australians, First Nations women were less likely to have received any of the recommended vaccines during pregnancy: dTpa; PR 0.70, 95%CI 0.69–0.71) and both vaccines; PR 0.69, 95%CI 0.67–0.71, and women from CALD backgrounds were more likely to have received IIV (PR 1.24, 95%CI 1.22–1.25) and both vaccines (PR 1.16, 95%CI 1.10–1.13) seen in Table 2.

**Table 2:** Prevalence of antenatal vaccine coverage among Links2HealthierBubs cohort, 2012–2017

Characteristics	*N (%)*	Total		
***a) IIV*** *(any)*	***69 670 / 441 747 (16)***		***PR (95% CI)***	***aPR (95% CI)***
Other Australians	48 196/320 008 (15)		*ref*	*ref*
First Nations	4 073/28 962 (14)		0.93 (0.91–0.95)	0.98 (0.96–0.99)
CALD	17 401/92 777 (19)		1.24 (1.22–1.25)	1.21 (1.20–1.23)
Age at infant birth < 20years	2 314/17 137 (14)		*ref*	*ref*
20–34 years	53 013/330 135 (16)		1.23 (1.17–1.28)	1.26 (1.20–1.32)
≥ 35 years	14 343/94 475 (15)		1.15 (1.09–1.20)	1.29 (1.23–1.36)
No 1st trimester antenatal care			*ref*	*ref*
Antenatal care in 1st trimester	50 283/283 670 (18)		1.44 (1.42–1.46)	1.50 (1.47–1.53)
Multiparous			*ref*	*ref*
Primiparous	39 278/ 198 348 (20)		1.25 (1.24–1.27)	1.26 (0.24–1.28)
Private hospital birth			*ref*	*ref*
Public hospital birth*	22 362/174 049 (13)		0.68 (0.66–0.68)	0.81 (0.80–0.83)
Northern Territory	1 976/18 433 (11)		*ref*	*ref*
Queensland	40 918/271 737 (15)		1.38 (1.33–1.44)	1.46 (1.41–1.52)
Western Australia	26 734/151 573 (18)		1.64 (1.58–1.71)	1.75 (1.68–1.82)
Year of infant birth 2012	2 008/74 797 (3)		*ref*	*ref*
2013	3 772/74 713 (5)		1.87 (1.79–1.96)	1.63 (1.51–1.77)
2014	3 654/72 791 (5)		1.81 (1.72–1.89)	1.07 (0.98–1.16)
2015	10 896/71 179 (15)		5.54 (5.32–5.76)	9.14 (8.56–9.76)
2016	22 092/75 854 (29)		10.49 (10.09–10.90)	18.90 (17.74–20.15)
2017	27 206/72 409 (38)		13.51 (13.02–14.06)	24.22 (22.73–25.81)
Not remote			*ref*	*ref*
Remote/Very remote	3 139/22 882 (14)		0.88 (0.85–0.90)	0.89 (0.86–0.91)
SEIFA 1 *n = 49 376*	6 816 (14)		*ref*	*ref*
SEIFA 2 *n = 44 082*	6 028 (14)		0.99 (0.95–1.03)	0.94 (0.90–0.97)
SEIFA 3 *n = 41 662*	6 223 (15)		1.10 (1.06–1.14)	0.96 (0.93-1.00)
SEIFA 4 *n = 42 891*	6 157 (14)		1.05 (1.01–1.09)	0.97 (0.93-1.00)
SEIFA 5 *n = 41 002*	6 382 (16)		1.15 (1.11–1.19)	0.98 (0.95–1.01)
SEIFA 6 *n = 45 341*	7 469 (16)		1.23 (1.19–1.28)	1.05 (1.02–1.09)
SEIFA 7 *n = 47 734*	8 151 (17)		1.29 (1.24–1.33)	1.07 (1.04–110)
SEIFA 8 *n = 44 852*	7 171 (16)		1.19 (1.15–1.23)	1.01 (0.98–1.04)
SEIFA 9 *n = 42 549*	7 597 (18)		1.36 (1.31–1.41)	1.10 (1.07–1.14)
SEIFA10 *n = 40 821*	7 372 (18)		1.38 (1.33–1.43)	1.17 (1.13–1.21)
***b) dTpa***† *(any)*	***n = 125 023 / 292 126 (43)***		***PR (95% CI)***	***PR (95% CI)***
Other Australians	90 726/207 560 (44)		*ref*	*ref*
First Nations	6 029/19 414 (31)		0.70 (0.69–0.71)	0.80 (0.79–0.82)
CALD	28 268/65 152 (43)		0.98 (0.97–0.98)	0.97 (0.96–0.98)
Age at infant birth < 20years	4 019/11 085 (36)		*ref*	*ref*
20–34 years	96 362/220 335 (44)		1.20 (1.17–1.22)	1.11 (1.09–1.14)
≥ 35 years	24 642/60 706 (41)		1.16 (1.13–1.19)	1.07 (1.04–1.09)
No 1st trimester antenatal care			*ref*	*ref*
Antenatal care in 1st trimester	90 186/194 414 (46)		1.19 (1.18–1.20)	1.15 (1.14–1.16)
Multiparous			*ref*	*ref*
Primiparous	71 593/143 236 (50)		1.24 (1.23–1.25)	1.25 (1.24–1.26)
Other birth facility			*ref*	*ref*
Public hospital birth*	55 763/115 949 (48)		0.90 (0.89–0.91)	0.97 (0.96–0.98)
Northern Territory	2 358/18 433 (13)		*ref*	*ref*
Queensland	88 765/271 737 (33)		2.54 (2.46–2.62)	2.44 (2.36–2.52)
Western Australia	33 811/151 573 (22)		1.74 (1.68–1.80)	1.67 (1.62–1.73)
Year of infant birth 2015	23 884/71 179 (34)		*ref*	*ref*
2016	48 070/75 854 (63)		1.91 (1.89–1.93)	1.37 (1.36–1.38)
2017	51 699/72 409 (71)		2.15 (2.13–2.18)	2.22 (1.12–4.72)
Not remote			*ref*	*ref*
Remote/Very remote	4 520/14 779 (31)		0.75 (0.73–0.76)	0.75 (0.74–0.77)
SEIFA 1 *n = 31 925*	11 203 (35)		*ref*	*ref*
SEIFA 2 *n = 28 264*	11 216 (40)		1.14 (1.12–1.16)	1.09 (1.07–1.10)
SEIFA 3 *n = 27 449*	11 378 (41)		1.19 (1.17–1.21)	1.12 (1.10–1.14)
SEIFA 4 *n = 27 731*	11 671 (42)		1.21 (1.19–1.23)	1.16 (1.14–1.18)
SEIFA 5 *n = 2 ,329*	12 117 (44)		1.25 (1.23–1.27)	1.20 (1.18–1.22)
SEIFA 6 *n = 30 346*	13 343 (44)		1.24 (1.22–1.25)	1.21 (1.19–1.23)
SEIFA 7 *n = 32 262*	14 691 (46)		1.28 (1.26–1.30)	1.22 (1.20–1.24)
SEIFA 8 *n = 29 811*	13 474 (45)		1.29 (1.27–1.31)	1.20 (1.18–1.22)
SEIFA 9 *n = 28 361*	13 078 (46)		1.30 (1.28–1.32)	1.21 (1.19–1.23)
SEIFA 10 *n = 27 351*	12 406 (45)		1.28 (1.26–1.30)	1.24 (1.22–1.26)
***c) Both***† *(IIV and dTpa)*	***n = 53 861/ 292 126 (18)***		PR* (95% CI)	PR* (95% CI)
Other Australians	37 967/207 560 (18)		*ref*	*ref*
First Nations	2 450/19 414 (13)		0.69 (0.67–0.71)	0.81 (0.78–0.84)
CALD	13 444/65 152 (21)		1.16 (1.10–1.13)	1.07 (1.06–1.09)
Age at infant birth < 20years	1 620/11 085 (15)		*ref*	*ref*
20–34 years	41 458/220 335 (19)		1.27 (1.22–1.32)	1.07 (1.02–1.13)
≥ 35 years	10 783/60 706 (18)		1.28 (1.23–1.34)	1.03 (0.97–1.08)
No 1st trimester antenatal care			*ref*	*ref*
Antenatal care in 1st trimester	39 727/194 414 (20)		1.30 (1.28–1.32)	1.21 (1.18–1.23)
Multiparous			*ref*	*ref*
Primiparous	32 094/143 236 (22)		1.33 (1.32–1.35)	1.34 (1.32–1.37)
Other birth facility			*ref*	*ref*
Public hospital birth*	19 202/115 949 (17)		0.63 (0.62–0.64)	0.68 (0.66–0.69)
Northern Territory	700/18 433 (4)		*ref*	*ref*
Queensland	35 658/271 737 (13)		3.40 (3.18–3.62)	3.36 (3.14–3.58)
Western Australia	17 469/151 573 (12)		2.97 (2.78–3.17)	2.95 (2.76–3.15)
Year of infant birth 2015	8 560/71 179 (12)		*ref*	*ref*
2016	20 129/75 854 (27)		2.21 (2.17–2.26)	2.20 (2.16–2.25)
2017	25 051/72 409 (35)		2.88 (2.83–2.94)	2.86 (2.80–2.91)
Not remote			*ref*	*ref*
Remote/Very remote	1 969/14 779 (13)		0.75 (0.72–0.78)	0.77 (0.74–0.79)
SEIFA 1 *n = 31 925*	4 817 (15)		*ref*	*ref*
SEIFA 2 *n = 28 264*	4 512 (16)		1.07 (1.04–1.11)	0.97 (0.94–1.01)
SEIFA 3 *n = 27 449*	4 774 (17)		1.15 (1.11–1.19)	1.01 (0.98–1.05)
SEIFA 4 *n = 27 731*	4 700 (17)		1.14 (1.11–1.18)	1.01 (0.98–1.05)
SEIFA 5 *n = 27 329*	5 035 (18)		1.20 (1.17–1.24)	1.04 (1.01–1.08)
SEIFA 6 *n = 30 346*	5 852 (19)		1.26 (1.23–1.30)	1.11 (1.07–1.15)
SEIFA 7 *n = 32 262*	6 467 (20)		1.33 (1.29–1.37)	1.12 (1.09–1.16)
SEIFA 8 *n = 29 811*	5 601 (19)		1.25 (1.21–1.29)	1.05 (1.02–1.09)
SEIFA 9 *n = 28 361*	6 054 (21)		1.40 (1.36–1.45)	1.16 (1.22–1.20)
SEIFA 10 *n = 27 351*	5 855 (21)		1.44 (1.40–1.48)	1.23 (1.19–1.27)

**Abbreviations:** PR, prevalence ratio; IIV, inactivated influenza vaccine; CALD, Culturally and linguistically diverse; SEIFA, Socio-Economic Indexes for Areas; dTpa, diphtheria-Tetanus-acellular pertussis vaccine

* Qld data only

†Data restricted to >2014 in line with recommendations for dTpa in pregnancy

All potential confounding variables included in the GLMM analysis are shown in **Table 2**. Factors that positively predicted antenatal vaccine coverage for the overall cohort were; increasing maternal age at birth, birthing in a private hospital, parity, attending antenatal care during the first trimester, and increasing socio-economic advantage (SEIFA) (Table 2). Factors positively associated with vaccine coverage within the population groups were similar to those overall, apart from increasing maternal age among First Nations women (PR 0.82, 95%CI 0.71–0.93), and relative to the other jurisdictions, antenatal vaccine coverage in the NT was consistently lower (Supplementary Table 2).

### Remoteness and socio-economic influence

Coverage of antenatal IIV was similar (13–19%) between population groups regardless of remoteness of living, however coverage of dTpa and both vaccines were 10–20% lower among First Nations women, particularly for those living remotely (Supplementary Fig. 1). This trend was consistent across each jurisdiction (Supplementary Fig. 2). Univariable and multivariable models confirmed statistically that overall, living remotely detracted from antenatal vaccination coverage: IIV PR 0.88 (95% CI 0.85–0.90); dTpa PR 0.75 (95% CI 0.73–0.76); and both vaccines PR 0.75 (95% CI 0.72–0.78) (Table 1). This trend remained consistent among all groups (Supplementary Table 2).

**Fig. 2:**
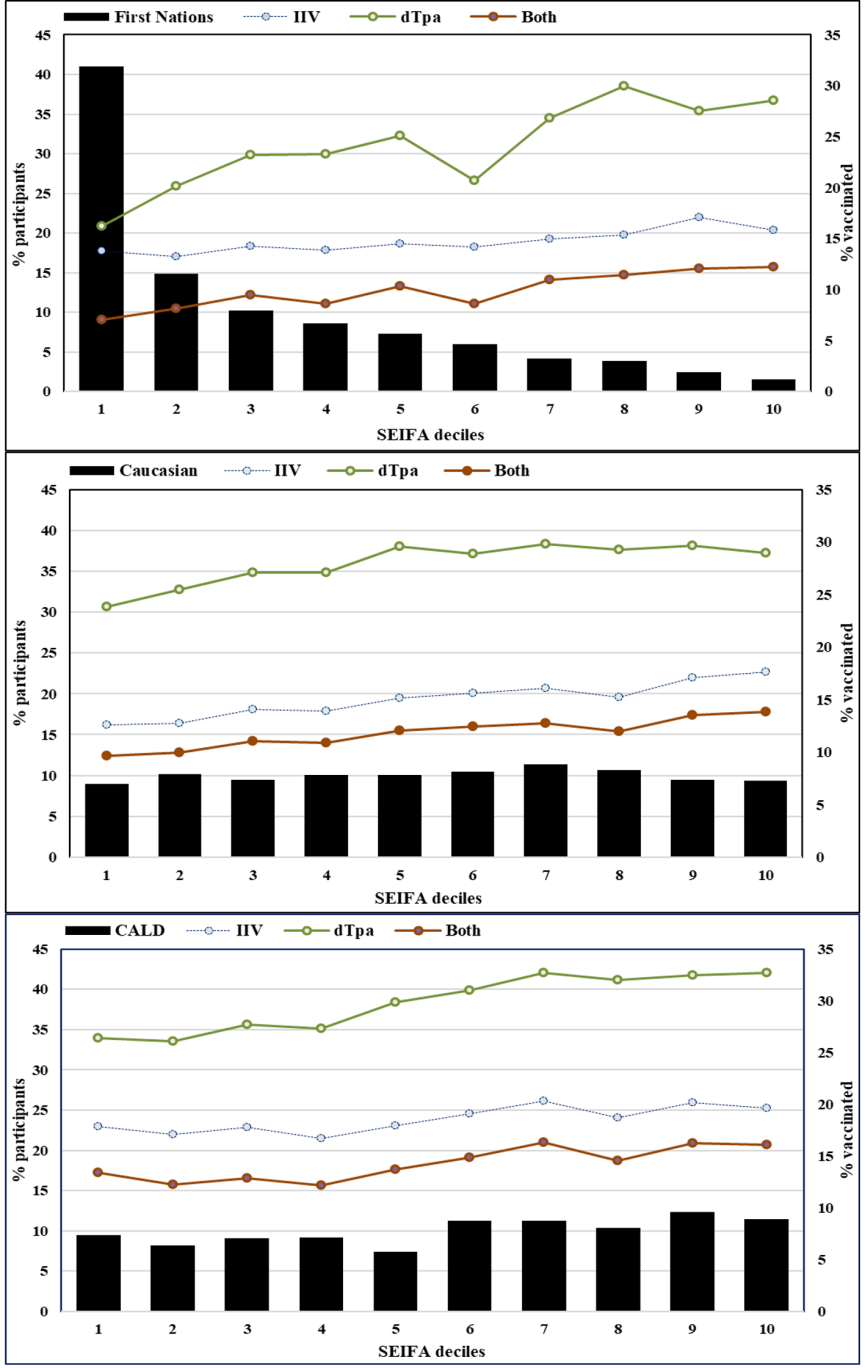
Antenatal vaccination by Indigenous status, ethnicity and socio-economic variation

Antenatal vaccine coverage increased with socio-economic advantage. The highest proportions of vaccine coverage were in the SEIFA 10 decile compared to women from areas of highest disadvantage (SEIFA deciles 1 and 2) shown in Fig. 2.

This positive association was most notable for coverage of dTpa, and both IIV and dTpa vaccines among First Nations women in SEIFA 9 decile: IIV (PR 1.28, 95%CI 1.05–1.57); dTpa, (PR 1.33, 95%CI 1.20–1.47); and both IIV and dTpa; (PR 1.36, 95%CI 1.14–1.62). The trends were consistent across each group (Supplementary Table 2), and each jurisdiction (supplementary Fig. 3a-c).

## Discussion

We identified overarching inequity in coverage of antenatal vaccinations among First Nations women, and in women who lived in remote areas, and women who lived in areas of higher socio-economic disadvantage. Other Australian women and CALD women living in major cities were more likely to have received the recommended and funded vaccines in pregnancy, and women from lower socio-economic backgrounds were less likely to have received vaccines. We also confirmed that younger women who birthed in public hospitals and who attended antenatal care later during pregnancy were less likely to have received antenatal vaccines. These geographic, social and financial access issues demonstrate shortcomings in our current health systems and NIP, and highlight the need for more dedicated resources and training to address the inequity.

As with other studies, we saw vaccination coverage improve over the years of the study, [28] particularly following the implementation of the antenatal dTpa program, but despite surveys indicating a high willingness of First Nations women to be vaccinated in pregnancy if offered, [10, 29] coverage of antenatal IIV and dTpa vaccination remained low. The gap between willingness and coverage is a potential physical and financial health systems access issue. Designing antenatal services specifically for younger mothers and promoting young parent programmes similar to some existing models may be one way to increase antenatal vaccination coverage among both younger and First Nations mothers [30]. Midwifery group practices co-designed with First Nations peoples for First Nations families, and antenatal care provided by First Nations health workers and midwives have demonstrated improved antenatal care attendance and equitable or better outcomes among First Nations mothers and infants compared to other Australians in mainstream care [31, 32]. However, this has not translated into practice for antenatal vaccination coverage in all jurisdictions as yet. Although our data relate to the years 2012–2017, data from a 2021 clinical audit within a major Qld public hospital showed both antenatal IIV and dTpa coverage remained considerably low among First Nations mothers (~ 17% for IIV and 26% for dTpa) compared to non-Indigenous mothers (39% for IIV and ~ 61% for dTpa) [33]. Other jurisdictions have also reported ongoing sub-optimal coverage of IIV after this time period (in 2017), with overall uptake ~ 48% in South Australia and Victoria, higher for dTpa (~ 79–83%), [34, 35] however, our data are the most comprehensive and contemporary for comparison thus far. An updated analysis of national data are warranted, particularly now that COVID-19 vaccination in pregnancy has been recommended since 2021 [36]. This recommendation may have influenced the priority of antenatal COVID-19 vaccination over antenatal IIV and dTpa administration during this time period, or pandemic restrictions may have affected access to antenatal IIV and dTpa.

Where First Nations or CALD focused midwifery models of care are not in place, more attention should be given to developing culturally safe clinical practice, and improving vaccine education and service delivery to avoid systemic barriers to equity such as racism and discrimination [37]. Strategies to increase antenatal vaccination coverage could include the use of appropriate language and interpreters in healthcare provider service settings.

### Strengths and limitations

Our data include the largest linked cohort of First Nations mother-infant pairs across three Australian jurisdictions, and includes urban, rural and remote-living representation. The WA validated algorithm used to identify maternal First Nations status across multiple datasets strengthens the accuracy of these data. Our data are also the first to describe antenatal vaccination status by areas of accessibility to services and socio-economic advantage and disadvantage based on individual postcode level data. Our antenatal vaccination coverage results are consistent with international studies conducted during similar time periods, with similar Indigenous and ethnic population proportions [6–8]. These studies have also suggested that sub-optimal antenatal IIV and dTpa vaccination will persist if strategies to address ongoing inequity are not met.

Our data did not include First Nations status or ethnicity of infants. We acknowledge that including Indigeneity only of the mother underrepresents First Nations infants of First Nations fathers, and that the mother may choose to not identify as First Nations due to risks of receiving poorer care [37]. Our large sample size of First Nations mothers however, affords good generalisability for the three jurisdictions included in our study. We also acknowledge potential limitations around using the variables ‘country of birth’ and ‘ethnicity’ as an imperfect way of assessing English language capacity and health literacy. We also acknowledge potential unknown errors of vaccine reporting.

## Conclusions

Our data demonstrated significant inequity in antenatal vaccination among First Nations Australian families, and families who live remotely and/or from lower socio-economic backgrounds. Given that the known increased risk factors for acquiring influenza or pertussis infections are living in remote regions, lower socio-economic status, inadequate housing, and limited access to culturally safe and appropriate, affordable health care, vaccination against these infections remains a key public health strategy in preventing severe disease in pregnancy and early infancy. Systematically monitoring vaccine coverage, and strategies that ensure vaccines are offered and provided to women equitably alongside other quality healthcare during pregnancy are required. This is a core responsibility of Australian health care systems and vaccine providers.

## Electronic supplementary material

Below is the link to the electronic supplementary material.

**Additional file 1: Supplementary box 1.** Vaccination sources by jurisdiction and year

**Additional file 2: Supplementary box 2.** Country of birth, Indigeneity and ethnicity of individual participants

**Additional file 3: Supplementary Table 1.** Indigeneity, ethnicity, remoteness and socio-economic advantage on antenatal vaccine coverage in Links2HealthierBubs cohort, 2012-2017

**Additional file 4: Supplementary Table 2.** Prevalence ratios of antenatal vaccine coverage among diverse population groups in the Links2HealthierBubs cohort, 2012-2017

**Additional file 5: Supplementary figure 1.** Vaccination status in pregnancy by Indigenous status and remoteness

**Additional file 6: Supplementary figure 2.** Vaccination status in pregnancy by Indigenous status and remoteness by jurisdiction

**Additional file 7: Supplementary Figure 3. a** Antenatal vaccination by Indigenous status, ethnicity, and socio-economic variation in NT. **b** Antenatal vaccination by Indigenous status, ethnicity, and socio-economic variation in Qld. **c** Antenatal vaccination by Indigenous status, ethnicity, and socio-economic variation in WA

## Data Availability

The datasets generated and/or analysed during the current study are not publicly available and are only made available to researchers through an application process. State and Territory Departments of Health and Data Custodians stipulate the non-sharing of these data, however analytic code used in this study can be made available upon request to the corresponding author.

## Abbreviations

IIV: inactivated influenza vaccines
dTpa: diptheria-tetanus-acellular pertussis vaccine
CALD: culturally and linguistically diverse
WA: Western Australia
NIP: National Immunisation Program
Links2HB: Links2HealthierBubs
NT: Northern Territory
Qld: Queensland
PDC: Perinatal Data Collections
IRSAD: Index of Relative Socio-economic Advantage and Disadvantage
SEIFA: Socio-Economic Indexes for Australia
ARIA: Accessibility Remoteness Index of Australia
GLMM: generalized linear mixed model
PR: prevalence risk
95% CIs: 95% compatibility intervals
aPR: adjusted prevalence ratios
