# Supplementary material for: Inequity of antenatal influenza and pertussis vaccine coverage in Australia: the Links2HealthierBubs record linkage cohort study, 2012–2017
Source: BMC Pregnancy Childbirth. 2023 May 8;23:314. doi: 10.1186/s12884-023-05574-w (PMC10164451; doi:10.1186/s12884-023-05574-w)
Supplement: Supplementary file 1 — Additional file 1: Supplementary box 1. Vaccination sources by jurisdiction and year [file 12884_2023_5574_MOESM1_ESM.docx]

**SUPPORTING INFORMATION**

**Supplementary box 1:** Vaccination sources by jurisdiction and year

| **Site** | **Northern Territory** | | **Queensland** | | **Western Australia** | |
| --- | --- | --- | --- | --- | --- | --- |
| **Dataset** | **NTIR** | **PDC** | **VIVAS** | **PDC** | **WAAVD** | **PDC** |
|  | | | | | | |
| Years available | 2011-2017 | - | 2011-2016 | 2015+ | 2011-2016 | 2016+ |
| Confirmed date/s of vaccination | ✓ | - | ✓ | - | ✓ | - |
| Self-reported vaccination | - | - | - | ✓ | - | ✓ |
| Gestation† | - | - | - | ✓ | ✓ | - |
| Trimester‡ | - | - | - | ✓ | ✓ | ✓ |

**Abbreviations:** NTIR, Northern Territory Immunisation Register; PDC, Perinatal data collection dataset; VIVAS, Vaccination Information and Vaccination Administration System; WAAVD, Western Australia Antenatal Vaccination Database.

† Gestation in weeks at time of vaccination in pregnancy

‡ Trimester of pregnancy at time of vaccination/s
