# Supplementary material for: Inequity of antenatal influenza and pertussis vaccine coverage in Australia: the Links2HealthierBubs record linkage cohort study, 2012–2017
Source: BMC Pregnancy Childbirth. 2023 May 8;23:314. doi: 10.1186/s12884-023-05574-w (PMC10164451; doi:10.1186/s12884-023-05574-w)
Supplement: Supplementary file 2 — Additional file 2: Supplementary box 2. Country of birth, Indigeneity and ethnicity of individual participants [file 12884_2023_5574_MOESM2_ESM.docx]

**SUPPORTING INFORMATION**

**Supplementary box 2:** Country of birth, Indigeneity and ethnicity of individual participants

| **Country of birth** | **N (%)** | **Ethnicity** | **N (%)** |
| --- | --- | --- | --- |
| Americas | 7,186 (1) | Caucasian | 105,138 (69) |
| Australia (incl.Oceania and Antarctica) | 310,732 (71) | Aboriginal and/or Torres Strait Islander | 6,227 (4) |
| New Zealand | 15,239 (4) | Asian | 17,044 (11) |
| North Africa and the Middle East | 6,509 (1) | Indian | 7,998 (5) |
| North-East Asia | 15,384 (3) | African/Negroid | 2,704 (2) |
| North-West Europe | 24,568 (6) | Polynesian | 340 (<1) |
| South-East Asia | 21,920 (5) | Maori | 2,410 (2) |
| Southern and Central Asia | 21,657 (5) | Other | 9,692 (6) |
| Southern and Eastern Europe | 5,676 (1) | **Total** | **151,553 (100)** |
| Sub-Saharan Africa | 12,349 (3) |  |  |
| **Total** | **441,220 (100)** |  |  |
