# Supplementary material for: Inequity of antenatal influenza and pertussis vaccine coverage in Australia: the Links2HealthierBubs record linkage cohort study, 2012–2017
Source: BMC Pregnancy Childbirth. 2023 May 8;23:314. doi: 10.1186/s12884-023-05574-w (PMC10164451; doi:10.1186/s12884-023-05574-w)
Supplement: Supplementary file 3 — Additional file 3: Supplementary Table 1. Indigeneity, ethnicity, remoteness and socio-economic advantage on antenatal vaccine coverage in Links2HealthierBubs cohort, 2012-2017 [file 12884_2023_5574_MOESM3_ESM.docx]

**SUPPORTING INFORMATION**

**Supplementary Table 1:** Indigeneity, ethnicity, remoteness and socio-economic advantage on antenatal vaccine coverage in Links2HealthierBubs cohort, 2012-2017.

| **Characteristics** | **First Nations**  N (%) | **Other Australian***  N (%) | **CALD**  N (%) |
| --- | --- | --- | --- |
| ***a) IIV*** *(any)* | ***4,073/28,962 (14)*** | ***48,196/320,008 (15)*** | ***17,401/92,777 (19)*** |
| Age at infant birth <20years | 762/5,132 (15) | 1,448/11,237 (13) | 104/768 (14) |
| 20-34 years | 2,944/20,885 (14) | 36,949/241,333 (15) | 13,120/67,917 (19) |
| ≥35 years | 367/2,945 (12) | 9,799/67,438 (15) | 4,177/24,092 (17) |
| Northern Territory | 623/5,823 (11) | 787/8,396 (9) | 566/4,214 (13) |
| Queensland | 2,225/15,912 (14) | 28,356/200,084 (14) | 10,337/55,741 (19) |
| Western Australia | 1,221/7,234 (17) | 19,041/111,423 (17) | 6,472/32,916 (20) |
| Year of infant birth 2012 | 393/4,835 (8) | 1,215/56,779 (2) | 400/13,183 (3) |
| 2013 | 265/4,668 (6) | 2,697/55,591 (5) | 810/14,454 (6) |
| 2014 | 228/4,798 (5) | 2,457/52,624 (5) | 969/15,369 (6) |
| 2015 | 534/4,819 (11) | 7,751/50,773 (15) | 2,611/15,587 (17) |
| 2016 | 1,139/5,064 (22) | 15,425/53,482 (29) | 5,528/17,308 (32) |
| 2017 | 1,510/4,785 (32) | 18,639/50,654 (37) | 7,057/16,970 (42) |
| Antenatal care in 1^st^ trimester | 2,515/15,225 (17) | 35,041/207,665 (17) | 12,727/60,780 (21) |
| Primiparous | 1,803/10,568 (17) | 27,545/142/362 (19) | 9,930/45,418 (22) |
| Public hospital birth§ | 2,001/14,799 (14) | 15,610/129,138 (12) | 4,751/30,112 (16) |
| Remote/Very remote | 1,289/9,909 (13) | 1,611/11,392 (14) | 239/1,581 (15) |
| SEIFA 1 *n=49,376* | 1,644/11,894 (14) | 3,613/28,683 (13) | 1,559/8,799 (18) |
| SEIFA 2 *n=44,082* | 575/4,267 (13) | 4,133/32,240 (13) | 1,320/7,575 (17) |
| SEIFA 3 *n=41,662* | 414/2,962 (14) | 4,312/30,327 (14) | 1,497/8,373 (18) |
| SEIFA 4 *n=42,891* | 334/2,466 (14) | 4,380/31,987 (14) | 1,443/8,438 (17) |
| SEIFA 5 *n=41,002* | 307/2,112 (15) | 4,829/31,994 (15) | 1,246/6,896 (18) |
| SEIFA 6 *n=45,341* | 259/1,734 (15) | 5,235/33,214 (16) | 1,975/10,393 (19) |
| SEIFA 7 *n=47,734* | 170/1,217 (14) | 5,856/36,132 (16) | 2,125/10,385 (20) |
| SEIFA 8 *n=44,852* | 179/1,087 (16) | 5,198/34,196 (15) | 1,794/9,569 (19) |
| SEIFA 9 *n=42,549* | 123/722 (17) | 5,166/30,360 (17) | 2,307/11,467 (20) |
| SEIFA10 *n=40,821* | 64/447 (14) | 5,248/29,812 (18) | 2,060/10,562 (20) |
| ***b) dTpa***† *(any)* | ***6,029/19,414 (31)*** | ***90,726/207,560 (44)*** | ***28,268/65,152 (43)*** |
| Age at infant birth <20years | 1,026/3,440 (30) | 2,825/7,726132 (40) | 168/513 (33) |
| 20-34 years | 4,469/14,063 (32) | 70,769/158,365 (45) | 21,124/47,907 (44) |
| ≥35 years | 534/1,911 (28) | 17,132/42,063 (41) | 6,976/16,732 (42) |
| Northern Territory | 489/5,823 (8) | 1,239/8,396 (15) | 630/4,214 (15) |
| Queensland | 4,374/15,912 (27) | 64,212/200,084 (32) | 20,179/55,741 (36) |
| Western Australia | 1,121/7,234 (16) | 25,141/111,423 (23) | 7,549/32,916 (23) |
| Year of infant birth 2015 | 985/4,819 (20) | 17,939/50,773 (35) | 4,960/15,587 (32) |
| 2016 | 2,248/5,064 (44) | 35,063/53,482 (66) | 10,759/17,308 (62) |
| 2017 | 2,689/4,785 (56) | 36,744/50,654 (73) | 12,266/16,970 (72) |
| Antenatal care in 1^st^ trimester | 3,708/10,731 (35) | 65,446/139,486 (47) | 21,032/44,197 (48) |
| Primiparous | 2,870/7,621 (38) | 52,117/101,993 (51) | 16,606/33,622 (49) |
| Public hospital birth§ | 4,033/10,029 (40) | 40,866/84,656 (48) | 10,864/21,264 (51) |
| Remote/Very remote | 1,492/6,490 (23) | 2,683/7,179 (37) | 345/1,110 (31) |
| SEIFA 1 *n=31,925* | 1,962/7,828 (25) | 6,914/18,016 (38) | 2,327/6,081 (38) |
| SEIFA 2 *n=28,264* | 881/2,769 (32) | 8,339/20,253 (41) | 1,996/5,242 (38) |
| SEIFA 3 *n=27,449* | 708/2,031 (35) | 8,320/19,561 (43) | 2,350/5,857 (40) |
| SEIFA 4 *n=27,731* | 581/1,628 (36) | 8,755/20,302 (43) | 2,335/5,801 (40) |
| SEIFA 5 *n=27,329* | 531/1,424 (37) | 9,509/21,008 (44) | 2,077/4,897 (42) |
| SEIFA 6 *n=30,346* | 361/1,167 (31) | 9,708/21,877 (44) | 3,274/7,302 (45) |
| SEIFA 7 *n=32,262* | 335/891 (38) | 10,942/23,978 (46) | 3,414/7,393 (46) |
| SEIFA 8 *n=29,811* | 339/784 (43) | 10,068/22,324 (45) | 3,067/6,703 (46) |
| SEIFA 9 *n=28,361* | 202/511 (40) | 9,084/19,805 (46) | 3,792/8,045 (47) |
| SEIFA 10 *n=27,351* | 125/339 (37) | 8,749/19,476 (45) | 3,532/7,536 (47) |
| ***c) Both***† *(IIV and dTpa)* | ***2,450/19,414 (13)*** | ***37,967/207,560 (18)*** | ***13,444/65,152 (21)*** |
| Age at infant birth <20 years | 428/3,440 (12) | 1,123/7,132 (16) | 69/513 (13) |
| 20-34 years | 1,818/14,063 (13) | 29,459/158,365 (19) | 10,181/47,907 (21) |
| ≥35 years | 204/1,911 (11) | 7,385/42,063 (18) | 3,194/16,732 (19) |
| Northern Territory | 158/5,823 (3) | 336/8,396 (4) | 206/4,214 (5) |
| Queensland | 1,646/15,912 (10) | 25,000/200,084 (12) | 9,012/55,741 (16) |
| Western Australia | 646/7,234 (9) | 12,581/111,423 (11) | 4,242/32,916 (13) |
| Year of infant birth 2015 | 345/4,819 (7) | 6,275/50,773 (12) | 1,940/15,587 (12) |
| 2016 | 941/5,064 (19) | 14,209/53,482 (27) | 4,979/17,308 (29) |
| 2017 | 1,158/4,785 (24) | 17,387/50,654 (34) | 6,506/16,970 (38) |
| Antenatal care in 1^st^ trimester | 1,566/10,731 (15) | 28,066/139,486 (20) | 10,095/44,197 (23) |
| Primiparous | 1,220/7,621 (16) | 22,818/101,993 (22) | 8,056/33,622 (24) |
| Public hospital birth§ | 1,473/10,029 (15) | 13,619/84,656 (16) | 4,110/21,264 (19) |
| Remote/Very remote | 618/6,490 (10) | 1,195/7,179 (17) | 156/1,110 (14) |
| SEIFA 1 *n=31,925* | 844/7,828 (11) | 2,791/18,016 (15) | 1,182/6,081 (19) |
| SEIFA 2 *n=28,264* | 356/2,769 (13) | 3,220/20,253 (16) | 936/5,242 (18) |
| SEIFA 3 *n=27,449* | 278/2,031 (14) | 3,391/19,561 (17) | 1,075/5,857 (18) |
| SEIFA 4 *n=27,731* | 206/1,628 (13) | 3,452/20,302 (17) | 1,042/5,801 (18) |
| SEIFA 5 *n=27,329* | 217/1,424 (15) | 3,866/21,008 (18) | 952/4,897 (19) |
| SEIFA 6 *n=30,346* | 152/1,167 (13) | 4,158/21,877 (19) | 1,542/7,302 (21) |
| SEIFA 7 *n=32,262* | 129/891 (14) | 4,654/23,978 (19) | 1,684/7,393 (23) |
| SEIFA 8 *n=29,811* | 132/784 (17) | 4,069/22,324 (18) | 1,400/6,703 (21) |
| SEIFA 9 *n=28,361* | 87/511 (17) | 4,085/19,805 (21) | 1,882/8,045 (23) |
| SEIFA 10 *n=27,351* | 47/339 (14) | 4,114/19,476 (21) | 1,694/7,536 (22) |

**Abbreviations:** CALD, Culturally and linguistically diverse; IIV, inactivated influenza vaccine; SEIFA, Socio-Economic Indexes for Areas; dTpa, diphtheria-Tetanus-acellular pertussis vaccine.

*Women who were Australian born, who did not identify as First Nations and were classified as ‘Caucasian’ in the variable ‘Ethnicity’

§ Qld data only; †Data restricted to >2014 in line with recommendations for dTpa in pregnancy
