# Supplementary material for: Inequity of antenatal influenza and pertussis vaccine coverage in Australia: the Links2HealthierBubs record linkage cohort study, 2012–2017
Source: BMC Pregnancy Childbirth. 2023 May 8;23:314. doi: 10.1186/s12884-023-05574-w (PMC10164451; doi:10.1186/s12884-023-05574-w)
Supplement: Supplementary file 4 — Additional file 4: Supplementary Table 2. Prevalence ratios of antenatal vaccine coverage among diverse population groups in the Links2HealthierBubs cohort, 2012-2017 [file 12884_2023_5574_MOESM4_ESM.docx]

**SUPPORTING INFORMATION**

**Supplementary Table 2:** Prevalence ratios of antenatal vaccine coverage among diverse population groups in the Links2HealthierBubs cohort, 2012-2017.

| **Characteristics** | **First Nations** | | **Other Australian*** | | **CALD** | |
| --- | --- | --- | --- | --- | --- | --- |
| ***a) IIV*** *(any)* | ***PR (95% CI)*** | ***aPR (95% CI)*** | ***PR (95% CI)*** | ***aPR (95% CI)*** | ***PR (95% CI)*** | ***aPR (95% CI)*** |
| Age at infant birth <20years | *ref* | *ref* | *ref* | *ref* | *ref* | *ref* |
| 20-34 years | 0.94 (0.86-1.03) | 0.91 (0.84-0.99) | 1.22 (1.16-1.29) | 1.01 (0.96-1.07) | 1.53 (1.24-1.88) | 1.46 (1.16-1.84) |
| ≥35 years | 0.82 (0.71-0.93) | 0.88 (0.78-1.00) | 1.15 (1.08-1.22) | 0.99 (0.93-1.05) | 1.34 (1.09-1.65) | 1.32 (1.05-1.67) |
| No 1^st^ trimester antenatal care | *ref* | *ref* | *ref* | *ref* | *ref* | *ref* |
| Antenatal care in 1^st^ trimester | 1.43 (1.36-1.50) | 1.28 (1.20-1.37) | 1.45 (1.42-1.47) | 1.12 (1.10-1.15) | 1.42 (1.38-1.46) | 1.14 (1.10-1.18) |
| Multiparous | *ref* | *ref* | *ref* | *ref* | *ref* | *ref* |
| Primiparous | 1.20 (1.14-1.26) | 1.28 (1.19-1.38) | 1.27 (1.25-1.28) | 1.28 (1.26-1.31) | 1.17 (1.14-1.20) | 1.17 (1.13-1.20) |
| Private hospital birth | *ref* | *ref* | *ref* | *ref* | *ref* | *ref* |
| Public hospital birth† | 0.70 (0.63-0.78) | 0.78 (0.70-0.86) | 0.66 (0.65-0.67) | 0.68 (0.66-0.69) | 0.72 (0.70-0.74) | 0.73 (0.71-0.76) |
| Northern Territory | *ref* | *ref* | *ref* | *ref* | *ref* | *ref* |
| Queensland | 1.27 (1.18-1.37) | 1.34 (1.24-1.44) | 1.49 (1.40-1.58) | 1.67 (1.57-1.77) | 1.37 (1.27-1.47) | 1.42 (1.32-1.53) |
| Western Australia | 1.56 (1.44-1.68) | 1.66 (1.53-1.80) | 1.82 (1.71-1.94) | 2.01 (1.89-2.13) | 1.46 (1.36-1.57) | 1.61 (1.50-1.74) |
| Year of infant birth 2012 | *ref* | *ref* | *ref* | *ref* | *ref* | *ref* |
| 2013 | 0.65 (0.57-0.74) | 0.26 (0.20-0.32) | 2.27 (2.15-2.41) | 2.31 (2.08-2.57) | 1.93 (1.74-2.15) | 2.88 (2.35-3.53) |
| 2014 | 0.57 (0.50-0.65) | 0.11 (0.08-0.16) | 2.14 (2.02-2.27) | 1.43 (1.27-1.60) | 2.04 (1.84-2.27) | 2.28 (1.85-2.81) |
| 2015 | 1.34 (1.21-1.49) | 1.13 (0.99-1.30) | 6.98 (6.63-7.34) | 13.68 (12.49-14.98) | 5.53 (5.04-6.06) | 14.04 (11.71-16.82) |
| 2016 | 2.64 (2.41-2.89) | 2.56 (2.29-2.87) | 13.13 (12.50-13.80) | 28.20 (25.79-30.84) | 10.57 (9.67-11.56) | 28.73 (24.04-34.33) |
| 2017 | 3.74 (3.43-4.08) | 3.04 (2.72-3.39) | 16.70 (15.89-17.54) | 36.12 (33.04-39.49) | 13.86 (12.69-15.14) | 37.30 (31.23-44.55) |
| Not remote | *ref* | *ref* | *ref* | *ref* | *ref* | *ref* |
| Remote/Very remote | 0.91 (0.86-0.96) | 0.89 (0.84-0.94) | 0.95 (0.91-0.99) | 0.96 (0.92-0.99) | 0.81 (0.73-0.90) | 0.83 (0.74-0.92) |
| SEIFA 1 *n=49,376* | *ref* | *ref* | *ref* | *ref* | *ref* | *ref* |
| SEIFA 2 *n=44,082* | 0.97 (0.88-1.08) | 0.92 (0.84-1.01) | 1.02 (0.97-1.07) | 0.99 (0.95-1.04) | 0.98 (0.90-1.06) | 0.84 (0.78-0.90) |
| SEIFA 3 *n=41,662* | 1.01 (0.90-1.14) | 0.95 (0.86-1.06) | 1.15 (1.10-1.21) | 1.03 (0.99-1.08) | 1.01 (0.93-1.09) | 0.84 (0.78-0.91) |
| SEIFA 4 *n=42,891* | 0.98 (0.86-1.11) | 0.91 (0.81-1.03) | 1.10 (1.05-1.15) | 1.05 (1.00-1.09) | 0.96 (0.89-1.04) | 0.81 (0.75-0.87) |
| SEIFA 5 *n=41,002* | 1.06 (0.95-1.21) | 0.94 (0.84-1.06) | 1.23 (1.18-1.29) | 1.05 (1.01-1.10) | 1.02 (0.94-1.11) | 0.86 (0.80-0.93) |
| SEIFA 6 *n=45,341* | 1.09 (0.95-1.26) | 0.86 (0.74-1.01) | 1.30 (1.24-1.36) | 1.12 (1.07-1.16) | 1.09 (1.01-1.17) | 0.94 (0.89-1.00) |
| SEIFA 7 *n=47,734* | 1.01 (0.85-1.20) | 0.88 (0.75-1.04) | 1.34 (1.28-1.40) | 1.12 (1.08-1.17) | 1.19 (1.11-1.28) | 0.98 (0.93-1.04) |
| SEIFA 8 *n=44,852* | 1.23 (1.04-1.45) | 0.96 (0.84-1.11) | 1.24 (1.19-1.30) | 1.07 (1.03-1.12) | 1.07 (0.99-1.16) | 0.90 (0.84-0.95) |
| SEIFA 9 *n=42,549* | 1.28 (1.05-1.57) | 0.93 (0.77-1.14) | 1.42 (1.36-1.49) | 1.18 (1.14-1.23) | 1.17 (1.09-1.26) | 0.96 (0.90-1.01) |
| SEIFA10 *n=40,821* | 1.04 (0.80-1.36) | 0.91 (0.71-1.16) | 1.48 (1.42-1.55) | 1.28 (1.23-1.33) | 1.13 (1.05-1.21) | 0.97 (0.91-1.02) |
| ***b) dTpa***§ *(any)* | ***PR (95% CI)*** | ***aPR (95% CI)*** | ***PR (95% CI)*** | ***aPR (95% CI)*** | ***PR (95% CI)*** | ***aPR (95% CI)*** |
| Age at infant birth <20years | *ref* | *ref* | *ref* | *ref* | *ref* | *ref* |
| 20-34 years | 1.04 (0.99-1.09) | 1.01 (0.96-1.07) | 1.12 (1.09-1.15) | 1.09 (1.06-1.12) | 1.33 (1.19-1.49) | 1.24 (1.10-1.40) |
| ≥35 years | 0.99 (0.92-1.07) | 0.95 (0.87-1.02) | 1.07 (1.04-1.10) | 1.05 (1.02-1.08) | 1.29 (1.16-1.44) | 1.18 (1.04-1.33) |
| No 1^st^ trimester antenatal care | *ref* | *ref* | *ref* | *ref* | *ref* | *ref* |
| Antenatal care in 1^st^ trimester | 1.20 (1.16-1.24) | 1.16 (1.12-1.21) | 1.15 (1.14-1.16) | 1.14 (1.13-1.15) | 1.27 (1.24-1.29) | 1.18 (1.16-1.20) |
| Multiparous | *ref* | *ref* | *ref* | *ref* | *ref* | *ref* |
| Primiparous | 1.31 (1.26-1.35) | 1.30 (1.25-1.34) | 1.23 (1.22-1.24) | 1.21 (1.20-1.22) | 1.24 (1.22-1.25) | 1.24 (1.22-1.25) |
| Private hospital birth | *ref* | *ref* | *ref* | *ref* | *ref* | *ref* |
| Public hospital birth† | 0.86 (0.81-0.91) | 0.92 (0.86-0.98) | 0.91 (0.90-0.92) | 0.97 (0.96-0.98) | 0.95 (0.94-0.97) | 1.02 (1.00-1.03) |
| Northern Territory | *ref* | *ref* | *ref* | *ref* | *ref* | *ref* |
| Queensland | 3.00 (2.79-3.23) | 3.03 (2.80-3.28) | 2.16 (2.07-2.276) | 2.21 (2.12-2.31) | 2.37 (2.23-2.52) | 2.41 (2.26-2.56) |
| Western Australia | 1.82 (1.67-1.97) | 1.85 (1.70-2.01) | 1.52 (1.45-1.59) | 1.54 (1.47-1.61) | 1.51 (1.41-1.61) | 1.55 (1.45-1.65) |
| Year of infant birth 2015 | *ref* | *ref* | *ref* | *ref* | *ref* | *ref* |
| 2016 | 2.13 (2.02-2.24) | 2.13 (2.10-2.25) | 1.89 (1.87-1.91) | 1.89 (1.87-1.91) | 1.96 (1.92-2.01) | 1.96 (1.92-2.01) |
| 2017 | 2.65 (2.52-2.79) | 2.62 (2.48-2.76) | 2.09 (2.07-2.11) | 2.08 (2.06-2.11) | 2.30 (2.25-2.35) | 2.29 (2.23-2.34) |
| Not remote | *ref* | *ref* | *ref* | *ref* | *ref* | *ref* |
| Remote/Very remote | 0.72 (0.69-0.75) | 0.76 (0.73-0.79) | 0.89 (0.87-0.91) | 0.88 (0.87-0.90) | 0.78 (0.72-0.84) | 0.79 (0.73-0.85) |
| SEIFA 1 *n=31,925* | *ref* | *ref* | *ref* | *ref* | *ref* | *ref* |
| SEIFA 2 *n=28,264* | 1.24 (1.18-1.31) | 1.04 (0.98-1.10) | 1.09 (1.07-1.11) | 1.10 (1.07-1.12) | 1.01 (0.97-1.05) | 1.01 (0.96-1.05) |
| SEIFA 3 *n=27,449* | 1.28 (1.21-1.36) | 1.04 (0.97-1.10) | 1.12 (1.10-1.14) | 1.13 (1.10-1.15) | 1.08 (1.04-1.12) | 1.10 (1.05-1.14) |
| SEIFA 4 *n=27,731* | 1.27 (1.19-1.35) | 1.08 (1.01-1.15) | 1.14 (1.12-1.16) | 1.17 (1.14-1.19) | 1.07 (1.03-1.11) | 1.06 (1.02-1.11) |
| SEIFA 5 *n=27,329* | 1.39 (1.31-1.48) | 1.15 (1.08-1.22) | 1.18 (1.15-1.20) | 1.20 (1.17-1.22) | 1.11 (1.07-1.16) | 1.11 (1.07-1.16) |
| SEIFA 6 *n=30,346* | 1.12 (1.04-1.22) | 1.16 (1.07-1.26) | 1.15 (1.13-1.17) | 1.20 (1.18-1.23) | 1.17 (1.13-1.21) | 1.14 (1.10-1.18) |
| SEIFA 7 *n=32,262* | 1.32 (1.22-1.43) | 1.17 (1.07-1.27) | 1.18 (1.16-1.20) | 1.21 (1.19-1.24) | 1.21 (1.17-1.25) | 1.16 (1.12-1.20) |
| SEIFA 8 *n=29,811* | 1.49 (1.38-1.60) | 1.17 (1.08-1.26) | 1.19 (1.17-1.21) | 1.20 (1.18-1.23) | 1.19 (1.15-1.23) | 1.13 (1.09-1.17) |
| SEIFA 9 *n=28,361* | 1.33 (1.20-1.47) | 1.09 (0.97-1.22) | 1.19 (1.17-1.21) | 1.21 (1.19-1.24) | 1.23 (1.19-1.27) | 1.15 (1.11-1.19) |
| SEIFA 10 *n=27,351* | 1.31 (1.17-1.48) | 1.20 (1.06-1.36) | 1.17 (1.15-1.19) | 1.23 (1.21-1.26) | 1.21 (1.18-1.26) | 1.18 (1.14-1.22) |
| ***c) Both***§ *(IIV and dTpa)* | ***PR (95% CI)*** | ***aPR (95% CI)*** | ***PR (95% CI)*** | ***aPR (95% CI)*** | ***PR (95% CI)*** | ***aPR (95% CI)*** |
| Age at infant birth <20years | *ref* | *ref* | *ref* | *ref* | *ref* | *ref* |
| 20-34 years | 1.01 (0.92-1.10) | 1.12 (1.00-1.25) | 1.17 (1.12-1.24) | 1.22 (1.15-1.30) | 1.56 (1.27-1.92) | 1.72 (1.32-2.25) |
| ≥35 years | 0.96 (0.84-1.10) | 1.13 (0.95-1.33) | 1.19 (1.13-1.26) | 1.26 (1.18-1.35) | 1.46 (1.18-1.79) | 1.64 (1.26-2.15) |
| No 1^st^ trimester antenatal care | *ref* | *ref* | *ref* | *ref* | *ref* | *ref* |
| Antenatal care in 1^st^ trimester | 1.32 (1.23-1.40) | 1.25 (1.16-1.35) | 1.27 (1.25-1.30) | 1.19 (1.16-1.22) | 1.31 (1.27-1.35) | 1.24 (1.22-1.30) |
| Multiparous | *ref* | *ref* | *ref* | *ref* | *ref* | *ref* |
| Primiparous | 1.36 (1.28-1.45) | 1.41 (1.30-1.53) | 1.33 (1.31-1.35) | 1.36 (1.33-1.38) | 1.27 (1.24-1.31) | 1.41 (1.30-1.53) |
| Private hospital birth | *ref* | *ref* | *ref* | *ref* | *ref* | *ref* |
| Public hospital birth† | 0.73 (0.65-0.82) | 0.78 (0.69-0.88) | 0.62 (0.61-0.64) | 0.67 (0.66-0.68) | 0.68 (0.66-0.70) | 0.73 (0.71-0.75) |
| Northern Territory | *ref* | *ref* | *ref* | *ref* | *ref* | *ref* |
| Queensland | 3.48 (3.02-4.00) | 3.50 (3.02-4.05) | 3.05 (2.78-3.35) | 3.38 (308-3.70) | 3.27 (2.89-3.69) | 3.36 (2.97-3.80) |
| Western Australia | 3.17 (2.74-3.67) | 3.27 (2.81-3.80) | 2.73 (2.49-3.00) | 3.00 (2.73-3.29) | 2.58 (2.28-2.92) | 2.87 (2.54-3.25) |
| Year of infant birth 2015 | *ref* | *ref* | *ref* | *ref* | *ref* | *ref* |
| 2016 | 2.48 (2.25-2.74) | 2.59 (2.30-2.91) | 2.16 (2.11-2.21) | 2.33 (2.26-2.40) | 2.34 (2.24-2.44) | 1.54 (1.19-2.00) |
| 2017 | 3.26 (2.96-3.58) | 3.06 (2.72-3.44) | 2.78 (2.72-2.84) | 3.03 (2.95-3.12) | 3.14 (3.01-3.27) | 1.39 (1.07-1.80) |
| Not remote | *ref* | *ref* | *ref* | *ref* | *ref* | *ref* |
| Remote/Very remote | 0.73 (0.68-0.79) | 0.72 (0.67-0.78) | 0.94 (0.90-0.98) | 0.95 (0.91-0.99) | 0.74 (0.65-0.85) | 0.77 (0.67-0.87) |
| SEIFA 1 *n=31,925* | *ref* | *ref* | *ref* | *ref* | *ref* | *ref* |
| SEIFA 2 *n=28,264* | 1.16 (1.05-1.28) | 0.95 (0.85-1.06) | 1.05 (1.01-1.09) | 1.02 (0.98-1.07) | 0.93 (0.87-1.00) | 0.85 (0.78-0.93) |
| SEIFA 3 *n=27,449* | 1.22 (1.10-1.35) | 0.98 (0.87-1.11) | 1.13 (1.09-1.17) | 1.06 (1.02-1.11) | 0.97 (0.91-1.03) | 0.87 (0.80-0.95) |
| SEIFA 4 *n=27,731* | 1.10 (0.98-1.24) | 0.93 (0.81-1.06) | 1.13 (1.09-1.18) | 1.08 (1.04-1.13) | 0.93 (0.87-0.99) | 0.81 (0.75-0.88) |
| SEIFA 5 *n=27,329* | 1.26 (1.12-1.42) | 1.02 (0.90-1.07) | 1.19 (1.14-1.23) | 1.09 (1.05-1.14) | 0.99 (0.92-1.06) | 0.90 (0.83-0.97) |
| SEIFA 6 *n=30,346* | 1.05 (0.91-1.21) | 0.89 (0.74-1.07) | 1.23 (1.18-1.28) | 1.16 (1.11-1.22) | 1.08 (1.02-1.15) | 0.96 (0.90-1.02) |
| SEIFA 7 *n=32,262* | 1.19 (1.03-1.38) | 0.97 (0.81-1.16) | 1.27 (1.22-1.31) | 1.16 (1.11-1.21) | 1.17 (1.11-1.24) | 1.01 (0.95-1.07) |
| SEIFA 8 *n=29,811* | 1.32 (1.14-1.53) | 0.99 (0.84-1.17) | 1.20 (1.16-1.25) | 1.10 (1.05-1.15) | 1.07 (1.00-1.13) | 0.91 (0.86-0.98) |
| SEIFA 9 *n=28,361* | 1.36 (1.14-1.62) | 1.01 (0.81-1.26) | 1.35 (1.30-1.40) | 1.22 (1.17-1.27) | 1.19 (1.12-1.26) | 0.98 (0.93-1.05) |
| SEIFA 10 *n=27,351* | 1.20 (0.96-1.51) | 1.02 (0.76-1.31) | 1.40 (1.35-1.45) | 1.32 (1.26-1.37) | 1.17 (1.11-1.24) | 1.00 (0.94-1.07) |

**Abbreviations:** Prevalence ratios; 95% CI, 95% compatability intervals; aPR; adjusted prevalence ratios, CALD, Culturally and linguistically diverse; IIV, inactivated influenza vaccine; SEIFA, Socio-Economic Indexes for Areas; dTpa, diphtheria-Tetanus-acellular pertussis vaccine.

*Women who were Australian born, who did not identify as First Nations and were classified as ‘Caucasian’ in the variable ‘Ethnicity’

†Qld data only

§Data restricted to >2014 in line with recommendations for dTpa in pregnancy
