# Supplementary material for: Inequity of antenatal influenza and pertussis vaccine coverage in Australia: the Links2HealthierBubs record linkage cohort study, 2012–2017
Source: BMC Pregnancy Childbirth. 2023 May 8;23:314. doi: 10.1186/s12884-023-05574-w (PMC10164451; doi:10.1186/s12884-023-05574-w)
Supplement: Supplementary file 6 — Additional file 6: Supplementary figure 2. Vaccination status in pregnancy by Indigenous status and remoteness by jurisdiction [file 12884_2023_5574_MOESM6_ESM.docx]

**SUPPORTING INFORMATION**

**
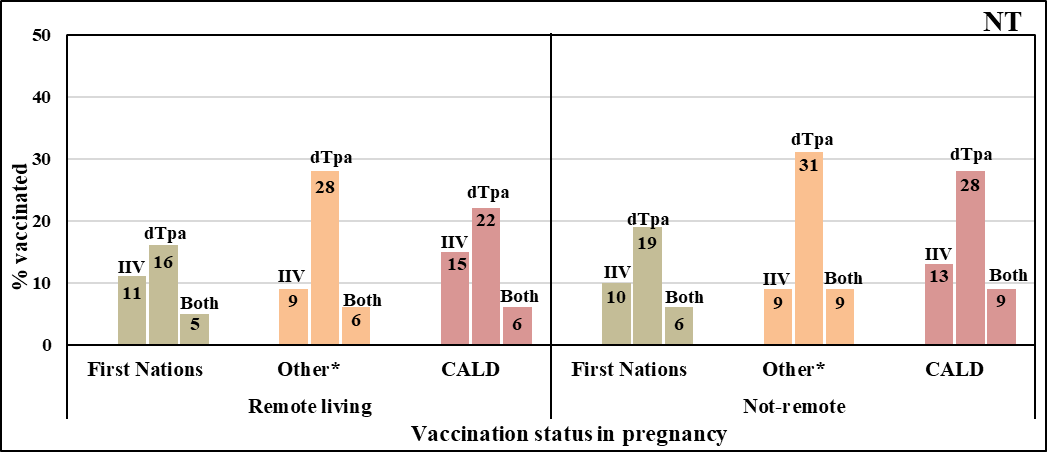
**

**
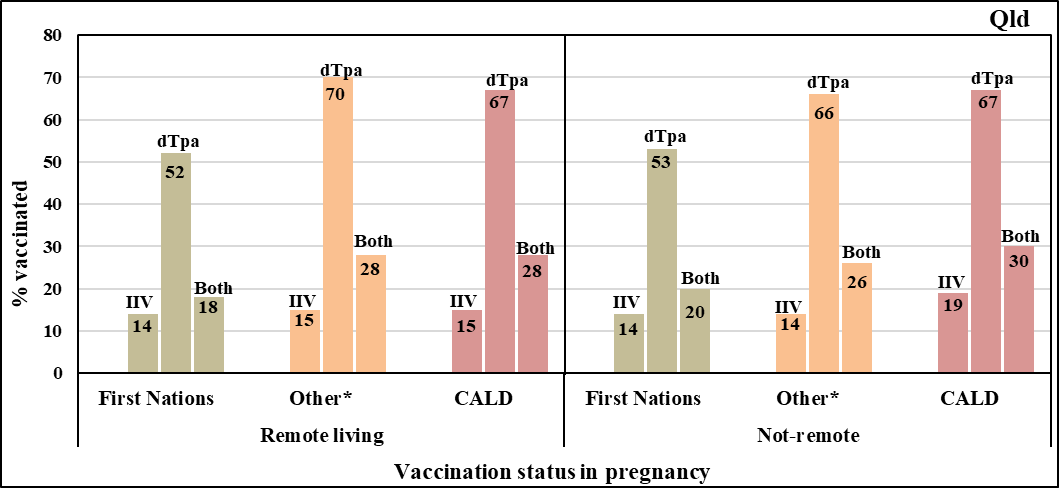
**

**
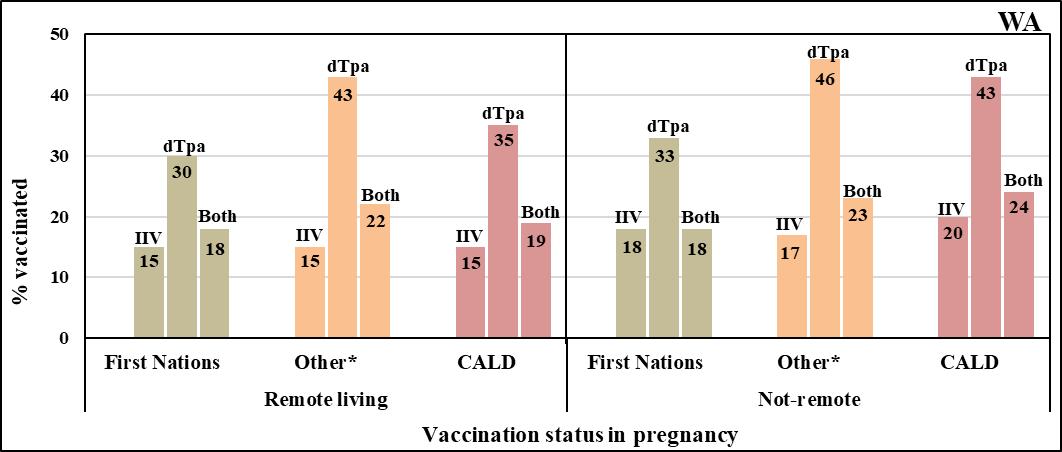
**

**Supplementary figure 2**: Vaccination status in pregnancy by Indigenous status and remoteness by jurisdiction

*Women who were Australian born, who did not identify as First Nations and were classified as ‘Caucasian’ in the variable ‘Ethnicity’
