# Supplementary material for: Inequity of antenatal influenza and pertussis vaccine coverage in Australia: the Links2HealthierBubs record linkage cohort study, 2012–2017
Source: BMC Pregnancy Childbirth. 2023 May 8;23:314. doi: 10.1186/s12884-023-05574-w (PMC10164451; doi:10.1186/s12884-023-05574-w)
Supplement: Supplementary file 7 — Additional file 7: Supplementary Figure 3. a Antenatal vaccination by Indigenous status, ethnicity, and socio-economic variation in NT. b Antenatal vaccination by Indigenous status, ethnicity, and socio-economic variation in Qld. c Antenatal vaccination by Indigenous status, ethnicity, and socio-economic variation in WA [file 12884_2023_5574_MOESM7_ESM.docx]

**SUPPORTING INFORMATION**


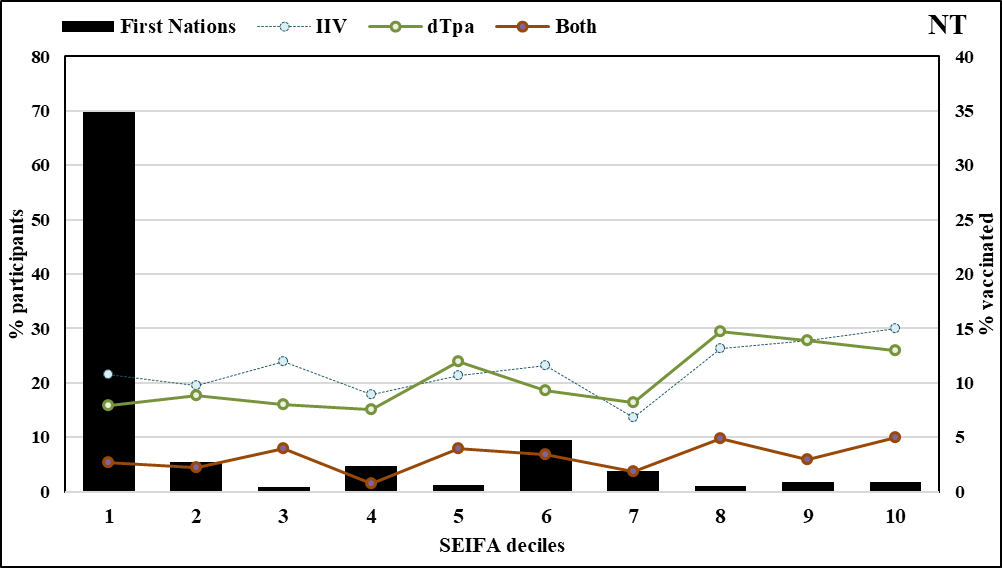

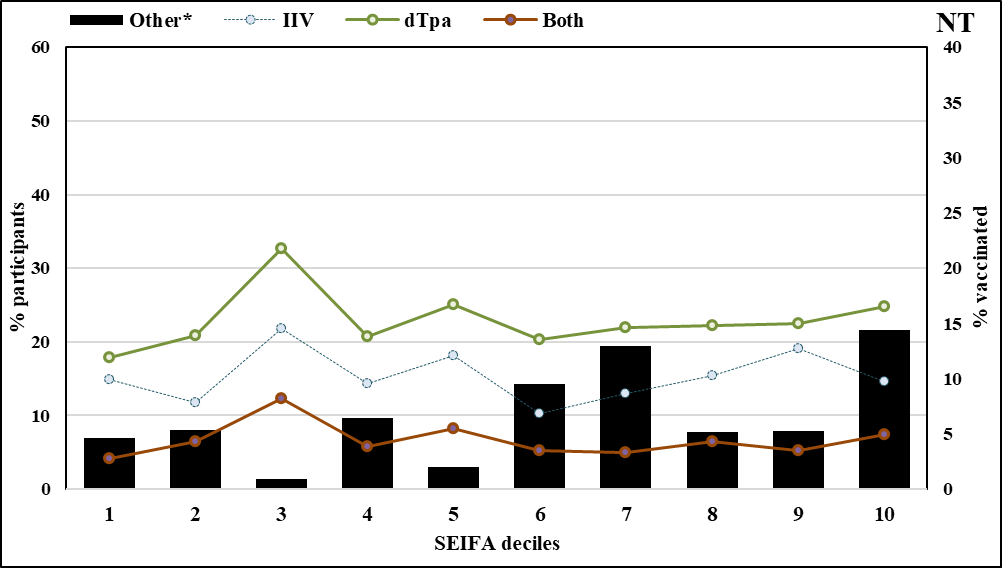

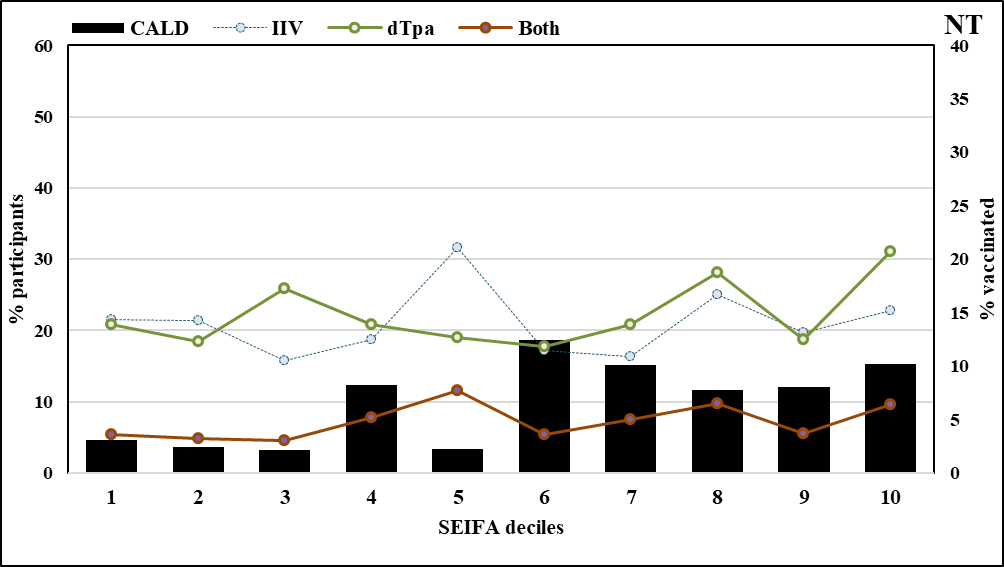
**Supplementary figure 3a**: Antenatal vaccination by Indigenous status, ethnicity, and socio-economic variation in NT

**
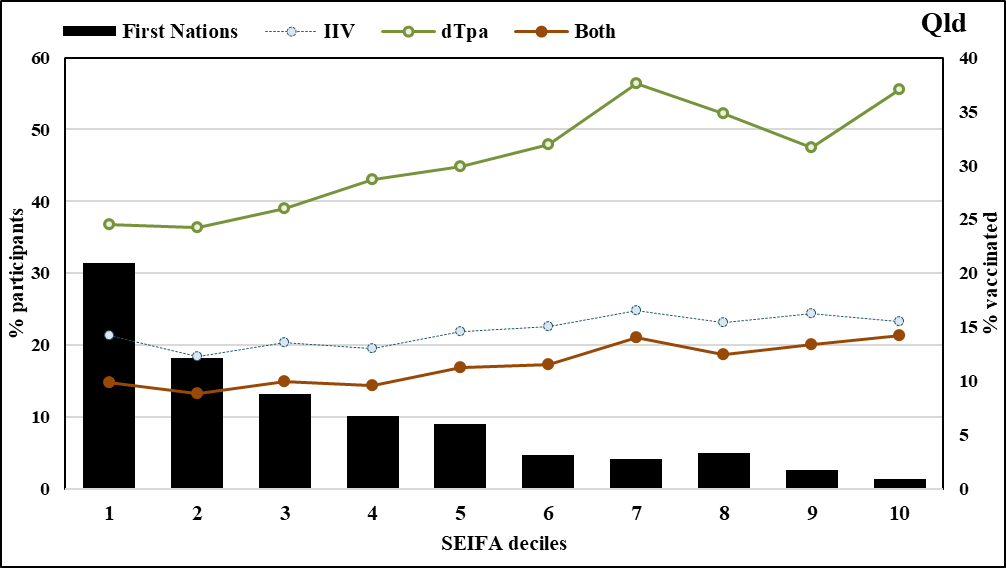
**

**
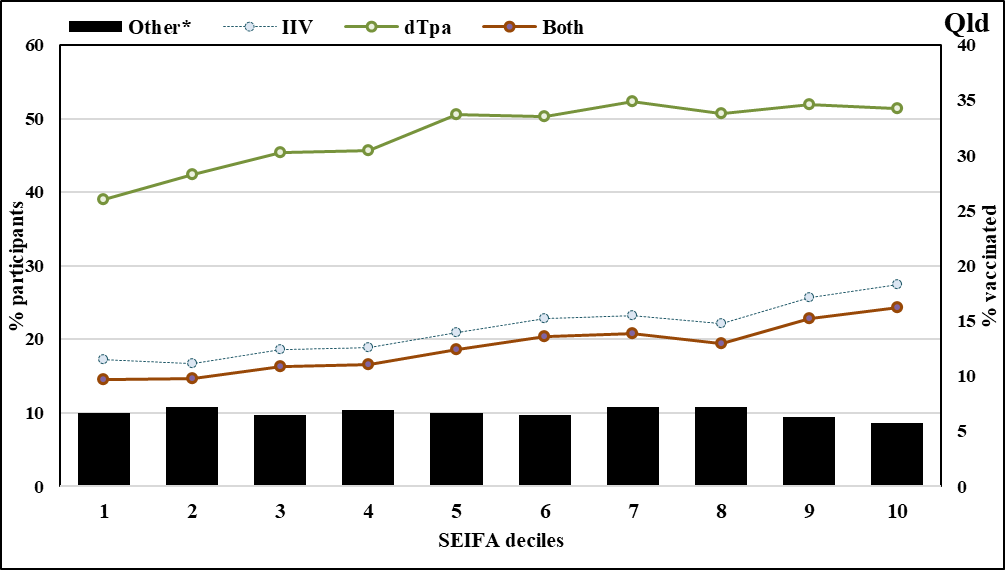
**


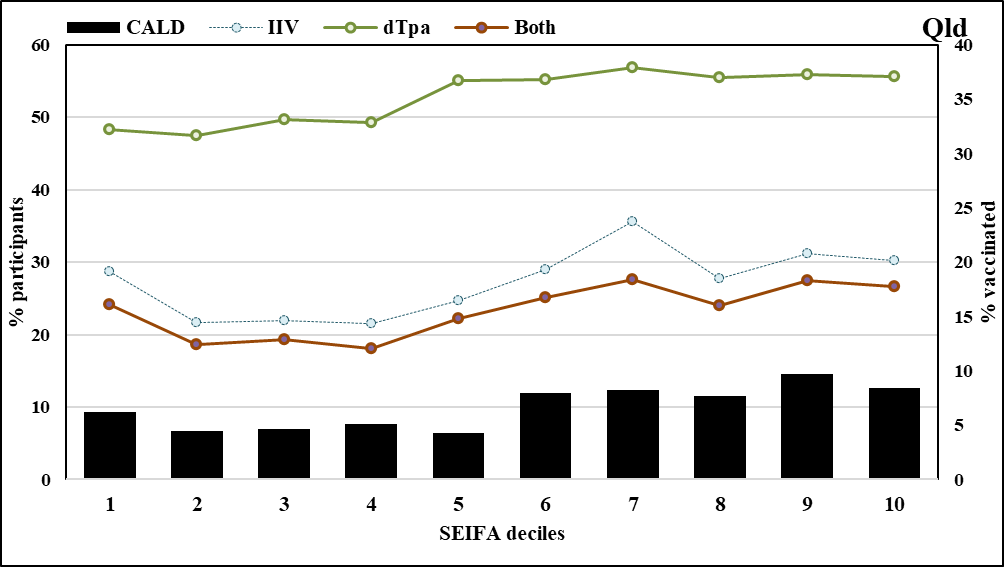


**Supplementary figure 3b**: Antenatal vaccination by Indigenous status, ethnicity, and socio-economic variation in Qld

**
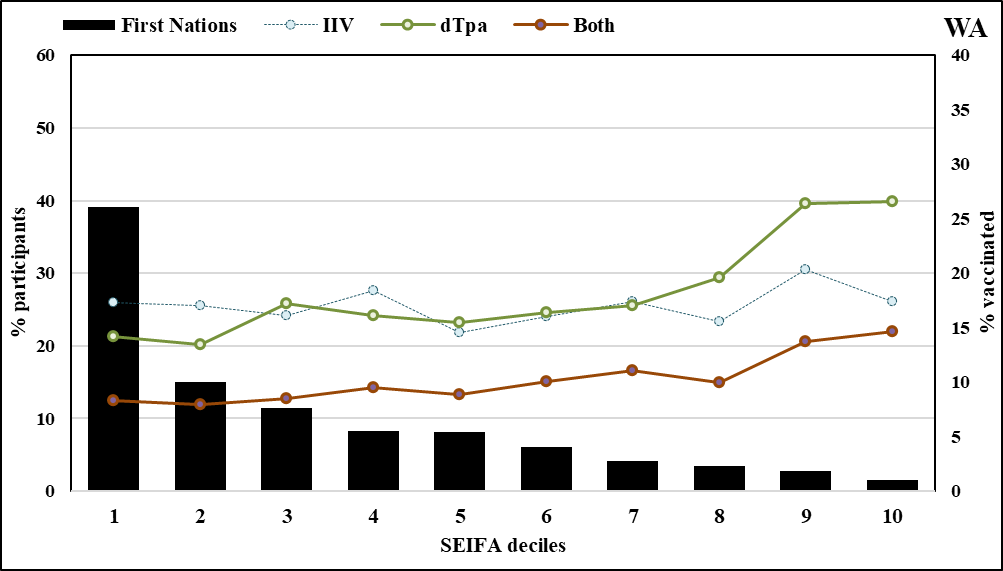
**

**
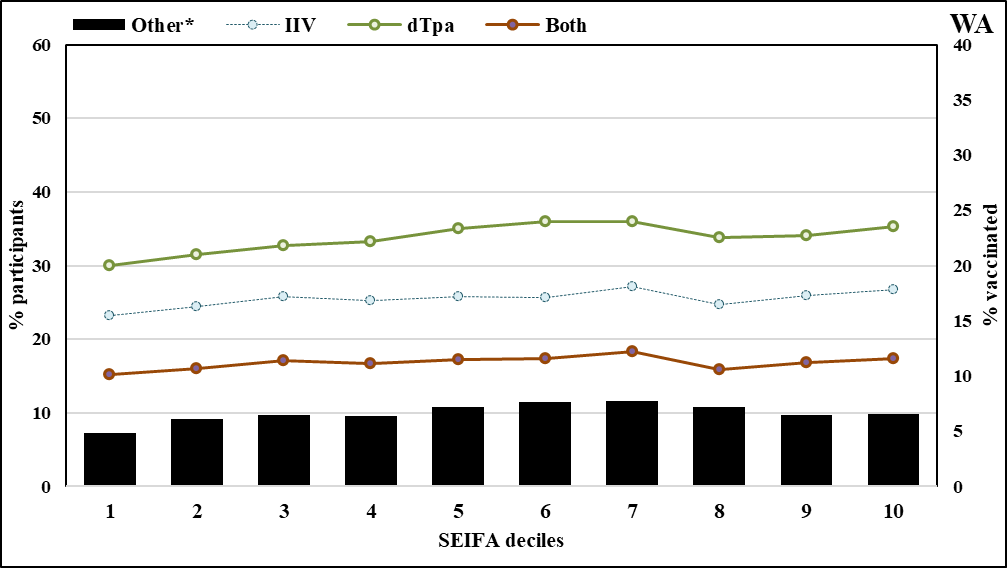
**


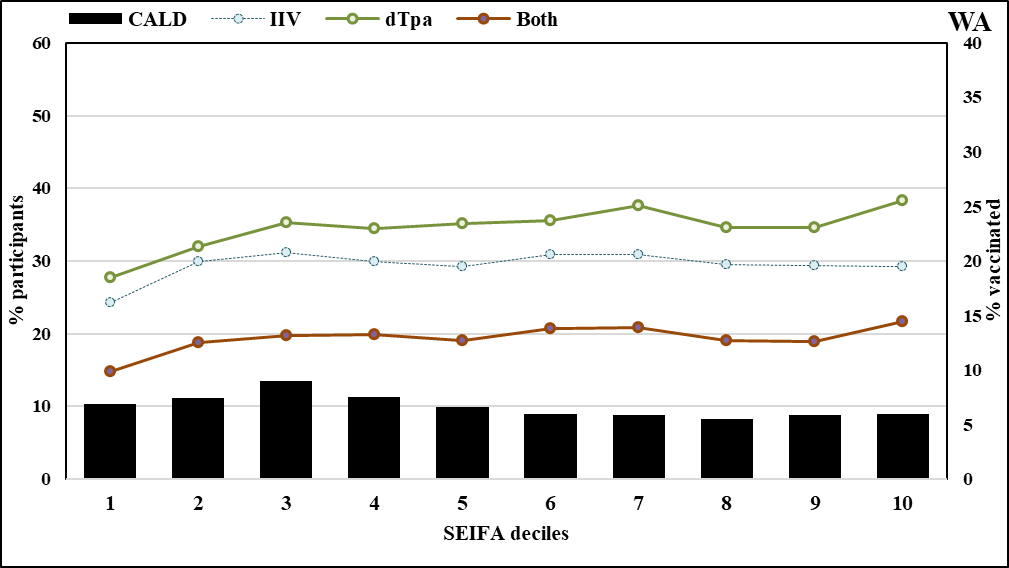


**Supplementary figure 3c**: Antenatal vaccination by Indigenous status, ethnicity, and socio-economic variation in WA

**ABBREVIATIONS**: CALD, Culturally and linguistically diverse; SEIFA, Socio-Economic Indexes for Australia;IIV, inactivated influenza vaccine; dTpa, diphtheria-tetanus-acellular pertussis vaccine

* Women who were Australian born, who did not identify as First Nations and were classified as ‘Caucasian’ in the variable ‘Ethnicity’
